# Characterization of Infections with Vancomycin-Intermediate *Staphylococcus aureus* (VISA) and *Staphylococcus aureus* with Reduced Vancomycin Susceptibility in South Korea

**DOI:** 10.1038/s41598-019-42307-6

**Published:** 2019-04-17

**Authors:** Jung Wan Park, Hyungmin Lee, Jung Wook Kim, Bongyoung Kim

**Affiliations:** 1 Department of Healthcare-Associated Infection Control, Korea Centers for Disease Control and Prevention, Cheongju, South Korea; 2 Department of Infectious Diseases, Asan Medical Center, University of Ulsan College of Medicine, Seoul, South Korea; 3 Division of Antimicrobial Resistance, Center for Infectious Diseases Research, Korea National Institute of Health, Korea Centers for Disease Control and Prevention, Cheongju, South Korea; 4 Department of Internal Medicine, Hanyang University College of Medicine, Seoul, South Korea

**Keywords:** Bacterial infection, Epidemiology

## Abstract

The aim of the present study was to describe the characteristics of infections with Staphylococcus aureus with reduced vancomycin susceptibility (SARVS) including vancomycin-intermediate S. aureus (VISA) in South Korea, using data from the national sentinel surveillance system during 2014–2016. A total of 66 patients infected or colonized with SA-RVS were reported using the sentinel surveillance system. Among them, VISA was confirmed in 14 isolates (21.2%) and no vancomycin-resistant S. aureus (VRSA) was detected. Most of patients had any kind of indwelling devices (81.8%, 54/66) and underwent surgical procedures in the previous 6 months (84.8%, 56/66). Patients who admitted to an intensive care unit (ICU) in the previous 3 months were 68.2% (45/66). Furthermore, patients who used vancomycin or had MRSA in the previous 1 month were 54.5% (36/66) and 59.1% (39/66), respectively. Upon review of the medical records, 54.5% (36/66) of patients were classified as having SA-RVSassociated infection and 30-day mortality was 19.4% (7/36). Our findings revealed that there was no VRSA in South Korea. SA-RVS including VISA existed particularly in patients who had indwelling devices, history of surgical procedure, and history of ICU admission.

## Introduction

Antimicrobial resistance (AMR) is an emerging global crisis and a present threat to public health^1^. Multidrug-resistant organisms (MDROs) increase mortality rate, length of hospital stay, and health care costs^2^. Medical costs attributable to infections caused by MDROs are estimated to range from $18,588 to $29,069 per patient in the United States^3^. In addition, potential cost savings have been estimated at $1,948 per patient if MDRO infection rates were reduced by 3.5%^4^. MDRO-related illness sometimes leads to legal conflicts as well^5^.

Unfortunately, MDROs are widespread throughout South Korea. According to the Korean Antimicrobial Resistance Monitoring System (KARMS), the proportions of methicillin-resistant *Staphylococcus aureus* (MRSA), vancomycin-resistant *Enterococcus faecium*, and carbapenem-resistant *Acinetobacter baumannii* were 66%, 30%, and 85%, respectively, in 2015^6^. Moreover, there have been substantial increases in the incidence of infections due to some MDROs, such as fluoroquinolone-resistant Enterobacteriaceae, vancomycin-resistant *E. faecium*, carbapenem-resistant *A. baumannii*, and carbapenem-resistant Enterobacteriaceae^6,7^. Consequently, some medical institutions in South Korea have experienced MDRO outbreaks^8,9^.

Among MDROs, vancomycin-intermediate *S. aureus* (VISA) is under special consideration by the Korea Centers for Disease Control and Prevention (KCDC). This is because infections caused by such pathogens leave clinicians with few therapeutic options for treatment.

The aim of the present study was to describe the characteristics of infections with *S. aureus* with reduced vancomycin susceptibility (SA-RVS) including VISA in South Korea, using data from Korea’s National Sentinel Surveillance System for VISA during 2014–2016.

## Material and Methods

### National Sentinel Surveillance for VISA

The KCDC started National Sentinel Surveillance for vancomycin-resistant *S. aureus* (VRSA) in 2000, and surveillance was expanded to SA-RVS in 2012.

All cases of *S. aureus* with vancomycin minimum inhibitory concentration (MIC) > 2 µg/mL using agar dilution, broth microdilution, or E-test, isolated at sentinel medical institutions, are mandatorily reported to the KCDC through the National Surveillance System for VISA. At the same time, sentinel medical institutions are required to send all isolates to the National Institute of Health (NIH) for further analysis. The sentinel medical institutions consist of all tertiary care hospitals (n = 44), all public hospitals (n = 39), and some hospitals with ≥200 beds (n = 17 in 2014 and 2015; n = 32 in 2016). These hospitals are distributed throughout the Korean Peninsula.

The KCDC performed in-depth surveillance for analysis of the current status of VISA dissemination from January 1, 2014 to December 31, 2016. On notification, the Epidemic Intelligence Service Officer of the KCDC (JW Park) reviewed the relevant medical records and gathered the following patient data: (i) demographic data, (ii) regional distribution, (ii) underlying comorbidities, (iii) risk factors for VISA acquisition, (iv) clinical features, (v) mortality, and (vi) admission history to other medical institutions. All cases were reviewed by staff of the KCDC department of Healthcare-Associated Infection Control and confirmed by the head of the department (H Lee). Both colonization and infection cases were included in this study.

### Laboratory procedures

At the laboratory of the NIH, confirmatory testing was performed for vancomycin MIC using agar dilution, broth microdilution, and E-test for all *S. aureus* isolates sent to the NIH. All isolates had MIC > 2 µg/mL by at least one of those methods and additional antimicrobial susceptibility testing and molecular typing were performed for them.

Antimicrobial susceptibility tests were conducted for penicillin, oxacillin, gentamicin, ciprofloxacin, clindamycin, erythromycin, telithromycin, linezolid, teicoplanin, tetracycline, tigecycline, nitrofurantoin, rifampicin, and trimethoprim/sulfamethoxazole, using the Vitek 2 (Biomérieux, Marcy I’Etoile, France). *S. aureus* ATCC 29213 was used as the quality control strain. For daptomycin susceptibility, an E-test was performed.

For molecular typing, genomic DNA was extracted using a Wizard Genomic DNA Purification Kit (Promega, Madison, WI, USA). According to the manufacturer’s protocol for bacterial cells, we added lysostaphin at a final concentration of 30 µg/mL in lysis buffer and incubated it at 37 °C for 1 h. Multilocus sequence typing (MLST) was carried out using polymerase chain reaction (PCR) amplification and sequencing of seven housekeeping genes (*arc*, *aroE*, *glpF*, *gmk*, *pta*, *tpi*, and *yqiL*) using primer pairs as previously described^10^. The allelic profiles and sequence types (STs) were assigned using the MLST website (http://saureus.mlst.net/). Staphylococcal Cassette Chromosome mec (SCC*mec*) types were identified with the multiplex PCR method^11^. Strains COL, N315, NCCP13860, and MW2 were included as controls for SCC*mec* types I, II, III, and IV, respectively. Amplification of the *spa* repeat region was performed using primers *spa*-1113f (5′-AAGACGATCCTTCGGTGAGC-3′) and *spa*-1514r (5′-CAGCAGTAGTGCCGTTTGCTT-3′). PCR products were sequenced and the *spa* types were determined using the Ridom SpaServer^12,13^. Gene responsible for the *vanA* or *vanB* were determined using PCR and DNA sequencing method as described previously^14,15^.

### Classification of *S. aureus* according to vancomycin MIC

Following the definition of the Clinical and Laboratory Standards Institute (CLSI), *S. aureus* isolates with vancomycin MIC 4–8 µg/mL were classified as VISA, and those with MIC ≥ 16 µg/mL were classified as VRSA^16^. The remaining reported *S. aureus* isolates, which showed vancomycin MIC between 2 and 4 µg/mL, were defined as non-VISA SA-RVS.

### Classification of patients with VISA or VRSA according to origin of the pathogen

Patients with VISA or VRSA were categorized as cases of “cross-transmission,” “present on admission,” or “sporadic” according to exposure history for the potential origin of the pathogen. A description of each category follows.

#### Cross-transmission

Isolation of VISA or VRSA which was identical with isolates from another patient who was hospitalized in the same ward within the 2 weeks. Isolates were considered to be genetically identical if the patterns of antibiogram, SCC*mec* type, ST, and *spa* type were identical^13^.

#### Present on admission

Isolation of VISA or VRSA within 48 hours of admission from patients who were transferred from other medical institutions.

#### Sporadic

isolation of VISA or VRSA within 48 hours of admission, and the category *Cross-transmission* or *Present on admission* was not applicable.

### Comparison between patients with VISA or VRSA and non-VISA SA-RVS

We performed a case-control study to identify differences in the characteristics of patients with VISA or VRSA compared to those with non-VISA SA-RVS, according to demographic data, regional distribution, underlying comorbidities, risk factors for VISA/VRSA acquisition, clinical categories, and mortality.

As for clinical categories, we defined patients who had compatible infectious signs and symptoms (such as bloodstream infection, urinary tract infection, pneumonia, surgical site infection, and so on) as having “SA-RVS-associated infection” based on the US Centers for Disease Control and Prevention/National Healthcare Safety Network (CDC/NHSN) surveillance definition of healthcare-associated infection^17^; the remaining cases were classified as “colonization with SA-RVS”.

### Descriptive study of fatal cases of SA-RVS-associated infection

To identify clinical characteristics of deaths caused by SA-RVS-associated infection, we performed a descriptive study. Demographic data, diagnosis on admission, risk factors for VISA or VRSA acquisition, and treatment regimen for SA-RVS-associated infections were summarized.

### Statistical analysis

All statistical analyses were conducted using SPSS version 21.0 for Windows (IBM Corp., Armonk, NY, USA). Categorical variables were analyzed using the chi-square test or Fisher’s extract test, as appropriate. Continuous variables were analyzed by independent *t*-tests. A two-tailed *P*-value of <0.05 was considered statistically significant.

### Ethics statement

The study protocol was approved by the Institutional Review Boards of the KCDC (2018-02-04-PE-A). The requirement for written informed consent from patients was waived due to the retrospective nature of the study and its impracticability.

## Results

**Figure 1:**
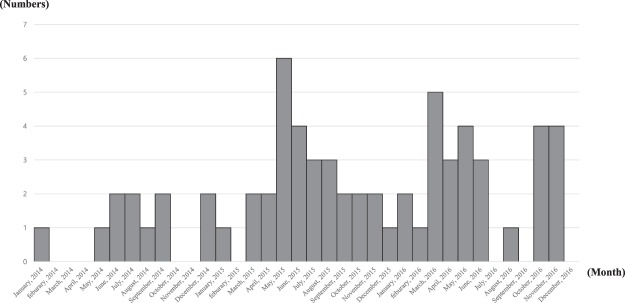
Number of reports for *Staphylococcus aureus* with reduced vancomycin susceptibility (SA-RVS) from sentinel medical institutions in South Korea, 2014–2016.

**Figure 2:**
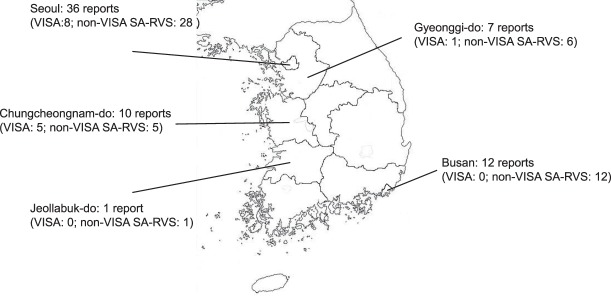
Geographic distribution of reported cases of *Staphylococcus aureus* with reduced vancomycin susceptibility (SA-RVS) in South Korea, 2014–2016.

A total of 66 patients infected or colonized with SA-RVS were reported using the sentinel surveillance system (11 in 2014, 28 in 2015, and 27 in 2016) (Fig. 1). The largest proportion of patients with SA-RVS was identified in Seoul (54.5%, 36/66), followed by Busan (18.2%, 12/66), Chungcheongnam-do (15.1%, 10/66), Gyeonggi-do (10.6%, 7/66), and Jeollabuk-do (1.5%, 1/66). No cases were identified in the other provinces (Fig. 2). As a result of confirmatory testing for vancomycin MIC, VISA was confirmed in 14 isolates (21.2%); no VRSA was detected. The MIC of the remaining 52 SA-RVS isolates (78.8%) was <4 µg/mL and their MIC were 3 µg/mL by E-test. Among VISA cases, 12 (85.7%, 12/14) were classified as “sporadic” cases, two (14.3%, 2/14) were classified as “present on admission,” and there were no cases of “cross-transmission”.

### Characteristics of patients carrying SA-RVS

Table 1 shows the characteristics of patients carrying SA-RVS. The mean age of patients was 62.3 ± 20.6 years and male sex predominated (72.7%, 48/66).

**Table 1:** Comparison between patients with vancomycin-intermediate *Staphylococcus aureus* (VISA) and non-VISA *S. aureus* with reduced vancomycin susceptibility (SA-RVS).

	Total (n = 66)	VISA (n = 14)	Non-VISA SA-RVS (n = 52)	*P*-value
Age (years), mean ± SD	62.3 ± 20.6	61.1 ± 21.6	62.7 ± 20.6	0.808
Male sex (%)	48 (72.7)	8 (57.1)	40 (76.9)	0.256
Underlying comorbidities (%)				
Diabetes	30 (45.5)	4 (28.6)	26 (50.0)	0.260
Liver diseases	8 (12.1)	0 (0.0)	8 (15.4)	0.269
Renal diseases	10 (15.1)	3 (21.4)	7 (13.5)	0.750
Any malignancy	15 (22.7)	3 (21.4)	12 (26.7)	0.841
Use of immunosuppressants	3 (4.5)	0 (0.0)	3 (5.8)	0.844
Risk factors for acquisition of SA-RVS (%)				
Use of indwelling devices	54 (81.8)	9 (64.3)	45 (86.5)	0.127
Surgical procedures in the previous 6 months	56 (84.8)	10 (71.4)	46 (88.5)	0.247
Admission to ICU in the previous 3 months	45 (68.2)	5 (35.7)	40 (76.9)	0.009
Vancomycin use in the previous 1 month	36 (54.5)	8 (57.1)	28 (53.8)	0.826
MRSA colonization in the previous 1 month	39 (59.1)	9 (64.3)	30 (57.7)	0.656
Clinical categories (%)				
SA-RVS-associated infection	36 (54.5)	6 (42.9)	30 (57.7)	0.492
Bloodstream infection	8 (12.1)	1 (16.7)	7 (23.3)	0.597
Urinary tract infection	1 (1.5)	0 (0.0)	1 (3.3)	0.833
Pneumonia	18 (27.3)	3 (50.0)	15 (50.0)	0.671
Surgical site infection	8 (12.1)	2 (33.3)	6 (20.0)	0.403
Others	1 (1.5)	0 (0.0)	1 (3.3)	0.833
Colonization with SA-RVS	30 (45.4)	8 (57.1)	22 (42.3)	0.322
Clinical outcomes (%)*				
In-hospital mortality	13/36 (36.1)	0/6 (0.0)	13/30 (43.3)	0.680
30-day mortality	7/36 (19.4)	0/6 (0.0)	7/30 (23.3)	0.317

Abbreviation: SD, standard deviation; MRSA, methicillin-resistant *Staphylococcus aureus*; ICU, intensive care unit.

*Denominator for patient groups with VISA and non-VISA SA-RVS is 6 and 30, respectively.

A 45.5% (30/66) of patients had diabetes and 22.7% (15/66) of them had any malignancy. Patients who had liver diseases and renal diseases were 12.1% (8/66) and 15.1% (10/66), respectively. Most of patients had any kind of indwelling devices (81.8%, 54/66) and underwent surgical procedures in the previous 6 months (84.8%, 56/66). Patients who admitted to an intensive care unit (ICU) in the previous 3 months were 68.2% (45/66). Furthermore, patients who used vancomycin or had MRSA in the previous 1 month were 54.5% (36/66) and 59.1% (39/66), respectively.

Upon review of the medical records, 54.5% (36/66) of patients were classified as having SA-RVS-associated infection. The most common infections were pneumonia (27.3%, 18/66), followed by bloodstream infection (12.1%, 8/66) and surgical site infection (12.1%, 8/66). Among patients with SA-RVS associated infection, 13 (36.1%, 13/36) patients died during admission and 30-day mortality was 19.4% (7/36).

When compared patients with VISA and patients with non-VISA SA-RVS, there was no significant difference in most parameters, except admission history to an ICU in the previous 3 months. It was significantly higher in patients with non-VISA SA-RVS than in patients with VISA (76.9% vs. 35.7%, *P* = 0.009).

As for clinical outcomes, there were no deaths among patients with VISA; however, 13 (43.3%, 13/30) patients with non-VISA SA-RVS died during admission; this difference was not statistically significant (*P* = 0.680).

### Deaths attributed to SA-RVS-associated infection

We analyzed patients who died within 30 days of hospital admission owing to SA-RVS-associated infections (Table 2). As shown in the table, all deaths occurred among patients with non-VISA SA-RVS.

**Table 2:** Clinical description of patients who died within 30 days owing to *S. aureus* with reduced vancomycin susceptibility (SA-RVS)-associated infections.

No.	Sex/Age (years)	Diagnosis on admission	SA-RVS-associated infection	Risk factors for acquisition of SA-RVS				Treatment regimen for SA-RVS-associated infection
				Admission to medical institution in past 3 months	Use of indwelling device	Surgical procedure in past 6 months	Admission to intensive care unit in past 3 months	
1	M/70	Aspiration pneumonia	Pneumonia	Y	Y	N	Y	Linezolid
2	M/69	Acute myelofibrosis	Pneumonia	Y	Y	N	Y	Vancomycin
3	F/47	Liver abscess, gallbladder cancer	Pneumonia	Y	Y	Y (percutaneous biliary drainage catheter insertion)	N	Linezolid
4	M/81	Aspiration pneumonia, bladder cancer	Bloodstream infection	Y	Y	N	Y	Linezolid
5	M/83	Aspiration pneumonia	Pneumonia	Y	Y	N	Y	N/A
6	M/81	Pan-peritonitis due to bowel perforation	Pneumonia	Y	Y	Y (small bowel resection)	Y	Teicoplanin
7	F/42	Hepatocellular carcinoma	Pneumonia	Y	Y	N	Y	N/A

The median patient age was 70 years, and five (71.4%, 5/7) patients were male. Among them, three patients (patient numbers 3, 4, and 7) had malignancies. Most patients (85.7%, 6/7) died owing to pneumonia. Nearly all patients had a history of admission to a medical institution in the previous 3 months (100.0%, 7/7), use of an indwelling device (100.0%, 7/7), and admission to an ICU in the previous 3 months (85.7%, 6/7). Two patients (28.6%, 2/7) had undergone a surgical procedure in the previous 6 months. Linezolid was administered to three patients for treatment of SA-RVS infection.

### Microbiological characteristics of VISA and non-VISA SA-RVS isolates

The *vanA* and *vanB* genes were not detected in any isolates. Regarding sample type, the largest proportion of SA-RVS was isolated from sputum (47.0%, 31/66), followed by nasopharyngeal swabs (16.7%, 11/66), and blood (10.6%, 7/66).

Table 3 shows the microbiological characteristics of VISA and non-VISA SA-RVS. The antimicrobial susceptibility of *S. aureus* isolates from patients with VISA to most antibiotics was similar to the susceptibility of isolates from patients with non-VISA SA-RVS. Both groups demonstrated high rates of susceptibility to linezolid, tigecycline, nitrofurantoin, and trimethoprim/sulfamethoxazole. VISA isolates were more susceptible than non-VISA SA-RVS isolates to penicillin (VISA: 14.3% vs. non-VISA SA-RVS: 0%, *P* = 0.042) and oxacillin (VISA: 14.3% vs. non-VISA SA-RVS: 0%, *P* = 0.042). On the other hand, the susceptibility rates of VISA isolates to teicoplanin (VISA: 69.2% vs. non-VISA SA-RVS: 94.2%, *P* = 0.025) and daptomycin (VISA: 85.7% vs. non-VISA SA-RVS: 100.0%, *P* = 0.042) were lower than those of non-VISA SA-RVS isolates.

**Table 3:** Microbiological characteristics of vancomycin-intermediate *S. aureus* (VISA) and non-VISA *S*. *aureus* with reduced vancomycin susceptibility (SA-RVS) isolates.

	Total (n = 66)	VISA (n = 14)	Non-VISA SA-RVS (n = 52)	*P*-value
Sample type (%)				
Blood	7 (10.6)	1 (7.1)	6 (11.5)	1.000
Nasopharyngeal swab	11 (16.7)	3 (21.4)	8 (15.4)	0.688
Sputum	31 (47.0)	4 (28.6)	27 (51.9)	0.120
Others	17 (25.7)	6 (42.8)	11 (21.1)	0.165
Antimicrobial susceptibility (%)				
Penicillin	2 (3.0)	2 (14.3)	0 (0.0)	0.042
Oxacillin	2 (3.0)	2 (14.3)	0 (0.0)	0.042
Gentamicin	23 (34.8)	7 (50.0)	16 (30.8)	0.215
Ciprofloxacin	4 (6.1)	1 (7.1)	3 (5.8)	1.000
Clindamycin	7 (10.6)	1 (7.1)	6 (11.5)	1.000
Erythromycin	4 (6.1)	0 (0.0)	4 (7.7)	0.571
Telithromycin	7 (10.6)	1 (7.1)	6 (11.5)	1.000
Linezolid	65 (98.5)	14 (100.0)	51 (98.1)	1.000
Teicoplanin	58 (87.9)	9 (69.2)	49 (94.2)	0.025
Tetracycline	9 (13.6)	2 (14.3)	7 (13.5)	1.000
Tigecycline	56 (84.8)	11 (78.6)	45 (86.5)	0.431
Nitrofurantoin	64 (97.0)	13 (92.9)	51 (98.1)	0.382
Rifampicin	33 (50.0)	9 (64.3)	24 (46.2)	0.367
Trimethoprim/sulfamethoxazole	66 (100.0)	14 (100.0)	52 (100.0)	—
Daptomycin	64 (97.0)	12 (85.7)	52 (100.0)	0.042
SCC*mec* (%)				
Type II	60 (90.9)	13 (92.9)	47 (90.4)	1.000
Type IV	6 (9.1)	1 (7.1)	5 (9.6)	1.000
Multilocus sequence typing (%)				
ST5	60 (90.9)	13 (92.8)	47 (90.4)	1.000
ST72	5 (7.6)	1 (7.1)	4 (7.7)	1.000
ST632	1 (1.5)	0 (0.0)	1 (1.9)	1.000
*spa* typing (%)				
t2460	38 (57.6)	7 (50.0)	31 (59.6)	0.518
t002	9 (13.6)	2 (14.3)	7 (13.5)	1.000
t324	5 (7.6)	1 (7.1)	4 (7.7)	1.000
t264	4 (6.1)	1 (7.1)	3 (5.8)	1.000
t9353	3 (4.5)	0 (0.0)	3 (5.8)	1.000
t686	2 (3.0)	0 (0.0)	2 (3.8)	1.000
t12703	1 (1.5)	1 (7.1)	0 (0.0)	0.212
t13420	1 (1.5)	1 (7.1)	0 (0.0)	0.212
t8444	1 (1.5)	0 (0.0)	1 (1.9)	1.000
Undefined	2 (3.0)	1 (7.1)	1 (1.9)	0.382

All isolates were either SCC*mec* type II or IV; SCC*mec* type II was the predominant isolate in both groups (VISA: 92.9% vs. non-VISA SA-RVS: 90.4%, *P* = 1.000). Compared with SCC*mec* type IV isolates, SCC*mec* type II isolates had significantly lower rates of susceptibility to gentamicin (type II: 30%, 18/60; type IV: 83.3%, 5/6; *P* = 0.030), ciprofloxacin (type II: 0.0%, 0/60; type IV: 66.7%, 4/6; *P* < 0.001), clindamycin (type II: 3.3%, 2/60; type IV: 83.3%, 5/6; *P* < 0.001), tetracycline (type II: 6.7%, 4/60; type IV: 83.3%, 5/6; *P* < 0.001), and rifampicin (type II: 48.3%, 29/60; type IV: 100.0%, 6/6; *P* = 0.039) (Table 4).

**Table 4:** Comparison of antimicrobial susceptibility of *S*. *aureus* with reduced vancomycin susceptibility (SA-RVS) isolates by SCC*mec* types.

	SCC*mec* II (N = 60)	SCC*mec* IV (N = 6)	*P*-value
Antimicrobial susceptibility (%)			
Penicillin	2 (3.3)	0 (0.0)	1.000
Oxacillin	2 (3.3)	0 (0.0)	1.000
Gentamicin	18 (30.0)	5 (83.3)	0.017
Ciprofloxacin	0 (0.0)	4 (66.7)	1.000
Clindamycin	2 (3.3)	5 (83.3)	1.000
Erythromycin	0 (0.0)	4 (66.7)	1.000
Telithromycin	2 (3.3)	5 (83.3)	1.000
Linezolid	59 (98.3)	6 (100)	1.000
Teicoplanin	52 (88.1)	6 (100)	1.000
Tetracycline	4 (6.7)	5 (83.3)	<0.001
Tigecycline	50 (83.3)	6 (100)	0.580
Nitrofurantoin	58 (96.7)	6 (100)	1.000
Rifampicin	27 (45.0)	6 (100)	0.024
Trimethoprim/sulfamethoxazole	60 (100)	6 (100)	—
Daptomycin	2 (3.3)	0 (0.0)	1.000

As for MLST, most isolates were ST5, comprising 92.8% (13/14) and 90.4% (47/52) of isolates from patients with VISA and non-VISA SA-RVS, respectively. The remaining isolates were ST72 (VISA: 7.1%, 1/14; non-VISA SA-RVS: 7.7%, 4/52; *P* = 1.000) and ST632 (VISA: 0.0%, 0/14; non-VISA SA-RVS: 1.9%, 1/52; *P* = 1.000).

As for *spa* typing, 9 known *spa* types were detected, with t2460 being the most prevalent (VISA: 50.0%, 7/14; non-VISA SA-RVS: 59.6%, 31/52; *P* = 0.518).

## Discussion

Herein, we report the characteristics of patients carrying SA-RVS including VISA in South Korea. Notably, no VRSA has been isolated in South Korea. The main mechanism for the occurrence of VRSA is via the presence of the *vanA* gene, which could be transferred from vancomycin-resistant enterococci to *S. aureus* via plasmids^15,18^. We found that no *S. aureus* isolates identified via the National Surveillance System for VISA had the *vanA* or *vanB* gene.

The presence of a thickened cell wall with an increased number of peptidoglycan layers is mostly applicable mechanism of reduced susceptibility to vancomycin among VISA^19^. The gene mutation that changes the pattern of the cell wall and/or reduces the expression of penicillin-binding proteins in *S. aureus* is triggered by long-term vancomycin usage^20,21^. In fact, the majority of VISA strains have emerged in patients with MRSA undergoing prolonged vancomycin therapy^19,20^.

Identified risk factors associated with VISA in previous studies include prolonged vancomycin use, previous MRSA colonization, hemodialysis dependence, and use of indwelling devices^19,22^. Furthermore, an observational study in the US revealed that prior vancomycin exposure within 30 days and residence in an ICU were predictors of SA-RVS presence in patients with *S. aureus* bacteremia^23^. Concordant with previous studies, we found that the majority of patients with SA-RVS used an indwelling device during hospitalization, had history of surgical procedures within 6 months, and admitted to ICU within 3 months.

There was no significant difference in most parameters including 30-day mortality between patients with VISA and patients with non-VISA SA-RVS. A previous study in the US suggested that patients with non-VISA SA-RVS infection had similar clinical characteristics compared to those with VISA infection^22^. Although elevated vancomycin MIC levels in patients with *S. aureus* infection may cause antibiotic therapy less effective, the association between vancomycin MIC and mortality is still controversial. Some previous meta-analyses have addressed association between vancomycin MIC and worse outcome^24,25^. On the contrary, no statistically significant difference in the mortality between patients with SA-RVS and those with non-SA-RVS were observed in the recent studies^26,27^. A prospective cohort study proposed that reduced vancomycin susceptibility might be linked to reduced disease severity in *S. aureus* infection^27^. Further studies are necessary to clarify this issue.

As for antimicrobial susceptibility, the SA-RVS strains appeared to have retained high susceptibility to linezolid, tigecycline, nitrofurantoin, and trimethoprim/sulfamethoxazole. Fortunately, these agents are available in South Korea and could be treatment options for SA-RVS-associated infections. When comparing VISA isolates with non-VISA SA-RVS isolates, the VISA strains were significantly less susceptible to teicoplanin. A previous report revealed that VISA is well correlated with lower response to teicoplanin because teicoplanin is a glycopeptide and has mechanisms similar to those of vancomycin^28^. Interestingly, two VISA isolates were resistant to daptomycin, which is not available in South Korea. Previous studies have revealed that increased vancomycin MIC in VISA isolates is associated with daptomycin resistance^29,30^. Sakoulas *et al*. suggested that exposure of *S. aureus* to vancomycin may affect daptomycin resistance^31^. In our study, two daptomycin-resistant VISA isolates had vancomycin MIC of 4 µg/mL. Another interesting finding was that two VISA isolates were susceptible to oxacillin whereas no non-VISA isolates were susceptible to this agent; both of these isolates carried SCC*mec* type II. Previous *in vitro* studies showed that decreased vancomycin susceptibility is associated with increased sensitivity to beta-lactams^32^. Based on such findings, the combination of vancomycin and beta-lactams has been suggested for treatment of SA-RVS infection^33^.

A recent retrospective study demonstrated that the molecular epidemiology of *S. aureus* is changing in Korea: a community-genotype MRSA strain, ST72, has emerged as a nosocomial pathogen^34^. Typically, SCC*mec* type II and ST5 strains are correlated with hospital-associated MRSA whereas SCC*mec* type IV and ST72 are correlated with community-acquired MRSA^35,36^. Despite the changing molecular epidemiology, SCC*mec* type II and ST5 remain predominant among SA-RVS strains in South Korea. Considering that most of our patients had risk factors for acquisition of SA-RVS, most SA-RVS strains might originate in health-care settings in South Korea.

Our study had some limitations. First, we could not acquire sufficient information about antibiotic usage. Basically, the in-depth surveillance in this study was mainly focused on the possibility of cross-transmission within medical institutions, and patients’ medical records from previous facilities were not fully available. Thus, we could not draw any conclusions about the relationship between the amount of vancomycin consumption and elevated vancomycin MIC in *S. aureus*. Second, most of the sentinel medical institutions are tertiary care hospitals and public hospitals. Therefore, the results of the present study are not generalizable to small- or medium-sized hospitals, including long-term care hospitals. Third, confirmatory testing for vancomycin MIC was performed by a variety of methods. E-test tends to yield higher numeric values for MIC than either broth or agar dilution and some isolates showed vancomycin MIC > 2 µg/mL only by E-test. It may have influenced data. Finally, the control group with fully vancomycin susceptible *S. aureus* was absent, which limits the ability to draw conclusions regarding clinical risk factors for VISA or SA-RVS acquisition.

Despite these limitations, the overall data of this study likely represent a reasonable approximation of the true values, and our findings may represent well the national status of SA-RVS in South Korea.

## Conclusion

Our findings revealed that there was no VRSA in South Korea. However, SA-RVS including VISA existed particularly in patients who had indwelling devices, history of surgical procedure, and history of ICU admission. Because only few therapeutic options exist and showing high mortality rate for infections caused by such pathogens, proper preventive strategies for further spread are indispensable. Implementing strict infection-control strategies and antimicrobial stewardship practices in each hospital is required in South Korea.

## Supplementary information

Dataset1
